# Experiences of peripartum depressive symptoms among Chinese middle-class migrant women in the Netherlands: a qualitative study of migrant motherhood

**DOI:** 10.1186/s12884-023-05957-z

**Published:** 2023-09-05

**Authors:** Haiyue Shan, Sawitri Saharso, Jens Henrichs

**Affiliations:** 1 Department of Sociology, Vrije University Amsterdam, De Boelelaan 1105, 1081HV, Amsterdam, Netherlands; 2 Amsterdam University Medical Center, Vrije University Amsterdam, Amsterdam, Netherlands

**Keywords:** Peripartum depressive symptoms, Navigating migrant motherhood, Healthy intimate relationships

## Abstract

**Background:**

A low educational level and poor economic status have repeatedly been identified as the main risk factors of peripartum depression among migrant women in existing studies. However, there is limited knowledge about a group of highly educated and middle-class migrant women, and how this group of migrant women deals with those risks and which protective factors facilitate a successful transition into motherhood in the host country. This study aims to shed light on the multifaceted psychosocial challenges during the peripartum period for Chinese migrant women in their relationships with intimate partners, mothers, and mothers-in-law.

**Methods:**

In this qualitative study, semi-structured in-depth interviews were conducted digitally with 46 pregnant and postpartum middle-class Chinese migrant women with peripartum depressive symptoms in the Netherlands. The interview data were analyzed using content analysis.

**Results:**

The multifaceted psychosocial challenges for women with peripartum depressive symptoms were classified into three key categories: the ambivalence towards different mothering values, perceived inadequate and mismatching social support and adverse childhood experiences.

**Conclusion:**

Well-educated middle-class Chinese migrant women with peripartum depressive symptoms faced challenges in the transition into motherhood due to the unmet self-expectations regarding the pursuit of a good quality of life and a happy motherhood. The nurturing intimate relationships and adequate social support in the host country have mitigated recollections of their adverse childhood experiences. Future prevention programs and postpartum care should consider the contextual specificity based on the childhood history. International mental health research should pay more attention to the growing and potentially vulnerable group of well-educated middle-class migrant women.

**Supplementary Information:**

The online version contains supplementary material available at 10.1186/s12884-023-05957-z.

## Introduction

Becoming a mother is always a major life event which starts in pregnancy and continues into the early postpartum period. This transitional process involves physical, hormonal changes and psychological changes. However, migrant mothers may experience additional difficulties in motherhood in foreign circumstances while acclimatizing to the host society [1, 2]. Their joy and well-being in pregnancy and motherhood are often intertwined with stress, vulnerability and frustration, due to their lower economic status, insufficient social support, the disruption of maternal rituals (e.g., *‘*doing the month*’* among Chinese mothers), and higher levels of anxiety related to migration [3]. This increases susceptibility to develop maternal mental health problems.

A growing amount of research suggests that migrant women have experienced particularly high rates of peripartum depression compared to native-born women [1]. Several studies indicate that the prevalence of postpartum depression during the first three months after delivery among Chinese women ranges from 10 to 20%, based on different assessment methods and the cultural characteristics of the population [4]. The adverse impacts of peripartum depression can range from poor maternal ability to meet the needs of newborns, reduction in affective involvement and sensitivity, to poor mother-infant bonding [5]. Many existing studies of the general population in Western countries have identified possible risk factors associated with peripartum depressive symptoms, including a history of depression, a lower educational level, a troubled marital relationship, a low level of family socio-economic status and a lack of social support [6–8].

Until now, the majority of research on risk factors associated with peripartum depression among migrant women has primarily focused on women with a low socio-economic status, and therefore, leaving a gap of our understanding regarding the specific vulnerability of middle-class migrant women [6, 9]. Certain class and education-based privileges can enhance migrant women’s capacities to acquire information and allocate resources for childcare tasks, which may potentially offer protection against developing peripartum depression during the pregnancy and the early postpartum stage. However, the challenges related to the transition to motherhood, and other stressors associated with migration and the adaptation to a new social context can simultaneously increase their vulnerabilities to develop peripartum depression [10]. As a result of globalization and digital communication, the characteristics of contemporary Chinese diaspora in the world have changed in the sense that the number of Chinese migrants who are higher educated and have a middle-class background is increasing [10, 11]. This trend is in particular visible among Chinese migrant women [12]. Therefore, we argue that existing explanations which focus on migrant women with a lower-class background cannot explain the prevalence of peripartum depression among the group of well-educated middle-class migrant women. Another knowledge gap in the literature is that, until now, the focus of peripartum depression related research has been on the identification of risk factors, but there is limited knowledge about how migrant women overcome those difficulties and which protective factors facilitate a successful transition into motherhood in the host country.

Moreover, migration itself is identified as one of the major stressors contributing to peripartum depression, as it can lead to separation from the woman’s own family and social networks and presents challenges to adjustment and integration to the new setting [7]. Among Chinese migrant women, social and interpersonal factors, such as poor relationships with husbands and mothers-in-law, and social demographic factors have been studied [9]. Additionally, practicing the custom of ‘doing the month’ might contribute to postpartum depression through restricting a mother’s liberty, undermining her autonomy, and reducing maternal confidence [13]. The traditional Chinese postpartum ritual of ‘doing the month’ (*zuo yuezi*) is meant to serve as a learning process for new mothers, and is also considered to protect women from developing postpartum depression after childbirth [14]. Historically and currently, women who have recently delivered newborns are regarded as vulnerable and weak. Therefore, they are expected to follow specific regulations to control their activities and diet, and they also receive social support from their mothers and mothers-in-law for the first month after childbirth [15, 16]. However, the strain and conflict between new mothers and in-laws in urban families often offsets the potential benefits of ‘doing the month’ [17].

In addition, the literature indicates that the younger generations of rising middle-class mothers in urban China are often beset by anxiety that is linked to the fear of failing in maternal responsibilities as well as uncertainties surrounding their children’s future [18]. Currently, Chinese middle-class parents rely upon experts in early childhood development for guidance concerning childcare standards [19]. This mothering norm among middle-class Chinese women echoes the neoliberal ideology of ‘intensive mothering,’ which originates in the West [20]. ‘Intensive mothering’ requires the mother to be the central caregiver, who puts children’s needs above hers. Women are encouraged to spend substantial resources, energy, and money in their children’s upbringing. According to the standards of intensive mothering, the obligation and burden of early childrearing mostly remain on women in urban China [21, 22].

In this study, we aim to address three research questions: 1) How middle-class Chinese migrant women with peripartum depressive symptoms experience the transition into motherhood in the Netherlands; 2)Which challenges and stressful circumstances are experienced by middle-class Chinese migrant women with peripartum depressive symptoms?, and 3)What support, enhancing their resilience, is experienced by middle-class Chinese migrant women with peripartum depressive symptoms? The findings of this study could equip healthcare professionals in the development of culturally sensitive maternal care to assist in dealing with the strenuous demands of early motherhood in transnational settings among the emerging group of higher educated migrant women.

## Methodology

### Design

This is a qualitative study focusing on middle-class Chinese migrant women in the Netherlands. Semi-structure in-depth interviews were conducted digitally from March 2020 to September 2021. Due to the COVID-19 regulations in the Netherlands since March 2020, recruitment and interviews were conducted digitally.

### Sampling and recruitment

The target population for the interviews was Chinese migrant (expectant) mothers in the Netherlands who had experienced or were experiencing peripartum depressive symptoms in their pre- and/or postpartum period. This time period ranged from approximately 24 weeks of pregnancy until six months postpartum. The migrants studied were immigrant Chinese women born and raised in the People’s Republic of China, Hong Kong, Macau, or Taiwan. Class information was not used as an inclusion criterion during the recruitment process, as the study’s original aim was to understand Chinese migrant women’s experiences in general. Based on the recruitment results, it was found that all participating women identified themselves as members of the middle class.

Chinese mothers and mothers-to-be living in various cities in the Netherlands were approached and invited to participate in this study by their midwives. Based on Dutch Central Bureau of Statistics population data, cities with the most Chinese residents were Rotterdam, Amsterdam, Den Haag, Eindhoven, and Utrecht. First, the interviewer the first author of this article) contacted all midwifery practices in these cities by sending an email invitation, and then followed up with a telephone call. The midwives forwarded the recruitment leaflet to potential participants with contact information for the interviewer. In addition, the recruitment leaflet and a video regarding this study were distributed in larger WeChat groups. Among Chinese migrants, the WeChat platform serves as a central hub for the Chinese diasporic media facilitating the maintenance of emotional ties of kinship and friendship with mainland China. By facilitating most formats of interpersonal communication, WeChat also enables the integration of Chinese migrants into a global digital diaspora that has become a source of emotional safety, especially during the COVID-19 pandemic [23]. Under the changing COVID restrictions, digital communication tools like WeChat could reach broader potential participants more efficiently. The snowball sampling approach was also used due to its ability to access hard-to-reach groups in sensitive topics. This approach emphasized the reliance on networking and social capital.

Participants were encouraged to initiate direct contact with the interviewer by email or text message. Before the interview, every woman completed the Edinburgh Postnatal Depression Scale (EPDS) or a Lifetime version of the EPDS. Women who scored 9 or higher were invited to an interview. Women with EPDS scores of 9 or higher were defined as exhibiting depressive symptomatology. This cut-off point has shown to have high sensitivity for detecting peripartum depression [24]. The EPDS score was used as a screening procedure in this study, but not for diagnosis and treatment purposes. Before an interview started, the interviewer explained the aims of the study and informed participants of their rights to refuse to continue with a portion of the study, or to end participation in the study overall. Afterwards, participants were asked to give written or oral consent to participate in this study [25, 26]. Among the 46 participating women, 31 granted written consent, while the remaining women provided oral consent before participating interviews. After providing consent, participants provided their demographic information by filling in a form or reporting the information orally to the interviewer. All the names used in the findings section are pseudonyms.

### Data collection and interviews

The interview topics ranged from the process of migrating to the Netherlands, pregnancy, childbirth, early childcare, the Chinese maternity tradition of *‘*doing the month*’ (zuo yuezi)*, challenges in the transition to motherhood to the amount of received social support. The question regarding how the participants understood their experiences of peripartum depression was asked when the woman actively started to share experiences of mood changes and emotional struggles. Due to the sensitivity of the interview topics, only two participants consented to audio recordings of their interviews. The interviewer took notes for the rest of the digital interviews. The duration of each interview was approximately 1.5 to 2.5 h.

Due to the ‘work from home policy’, many participating women had difficulty reserving longer time periods to complete the full interview in one instance. As a consequence, the interviewer sent two or three questions at a time. Then emerging themes were followed up in a successive interview session. The conversational characteristics of digital communication helped reduce the pressure on participants and was less intrusive than face-to-face interviews. The text-based nature of this method offered a degree of anonymity that might lower the discomfort and embarrassment among participants. In addition, a longer response time of sending text messages allowed participants to reflect and construct their narratives, especially concerning personal and sensitive experiences. All interviews, including text and voice messages, were transcribed in Chinese on the day they were taken and translated in English by the first author of this article. Another researcher being proficient in the Chinese was involved in the translation process to validate the accuracy of the translation.

### Coding and analysis

Content analysis in Mandarin was used to analyze the interviews. Data analysis consisted of two sequential stages of initial and focused coding, respectively. The process began by creating a broader narrative profile describing each participant’s mothering context in order to grasp the diverse conditions in each family. The profiles included details of the date of migration, reasons for migration and any transnational marriage. The initial codes employed line-by-line open coding and the process aimed to gain a broad understanding of each subject. Parallel to this, extensive memo writing was employed to help the authors reflect on any potential biases in the coding process. At the second stage of coding, the interviews were re-coded to identify general categories and patterns. After that, key themes, general patterns, and categories with examples of quotes were discussed among the co-authors. Several key themes became salient at this stage, including maternal roles and ‘intensive mothering,’ friction between Dutch and Chinese mothering ideals, adverse childhood experiences, healthy intimate relationships, and deficient social support from mothers and mothers-in-law.

## Results

A total of 46 pregnant and postpartum Chinese migrant women participated in-depth interviews. Table 1 presents information on the participants’ characteristics. On average, the 46 participants were 34 years old (age range: 25 to 43 years). The number of transnational couples with Dutch partners was 28 (61%) and the remaining 18 (39%) couples were both Chinese. The majority of participants were highly educated, i.e., more than half (52%) of the women had obtained a master’s degree. At the time of their pregnancy, half of the participants were housewives, and 15 (33%) women held a full-time job. All Chinese women in our study have adopted the Chinese maternity tradition of *‘*doing the month*’ (zuo yuezi)* and most of them (80%) have received help and support from their mothers or mothers-in-law during the critical one month after birth.

**Table 1:** Demographic details of participants

Characteristics	Variables	Numbers (%)
The age group (years)	20 ≤ 30	6 (13%)
	30 ≤ 40	36 (78%)
	40 ≤ 50	4 (9%)
Transnational couples	Yes	28 (61%)
	No	18 (39%)
Highest Educational Attainment	High vocational college	2 (4%)
	Undergraduate degree	17 (37%)
	Master’s degree	24 (52%)
	Doctoral degree	3 (7%)
Employment status at the time of giving birth	Full-time Part-time Not employed	15 (33%) 7 (15%) 24 (52%)
Reasons of migration	Education	22 (48%)
	Family	15 (33%)
	Employment	9 (19%)
The length of migrating to the Netherlands at the time of the birth	Less than a year	4 (9%)
	1–5 years	25 (54%)
	5–10 years	14 (30%)
	10 years and above	3 (7%)
Whether the mother/mother-in-law visited during early postpartum	Yes	37 (80%)
	No	9 (20%)

The average score on EPDS or the Lifetime version of EPDS was 11.5. The majority of women expressed mild depressive symptoms and anxiety during the early postpartum while several women (20%) had an EPDS score above 13 indicating clinical levels of depressive symptomatology with varying severity and were suggested to seek for professional help by the interviewer. However, only 3 women had visited therapists and were diagnosed with mild peripartum depression in the past months as reported by the women.

## Findings

The multifaceted psychosocial challenges experienced by middle-class Chinese migrant women with peripartum depressive symptoms were classified into three main categories: the ambivalence towards different mothering values, perceived inadequate and mismatching social support and adverse childhood experiences. Key themes under each category are presented in Table 2.

**Table 2:** Main categories and key themes of psychosocial challenges among middle-class Chinese migrant women with peripartum depressive symptoms

Category	Themes
The ambivalence towards different mothering values	-Perceived social pressure in exclusive breastfeeding
	-Fear of not reaching the standards of “intensive mothering”
	- The ambivalence of adopting different parenting styles
Perceived inadequate and mismatching social support	-The intergenerational differences in postpartum care and childcare
	-The tension and conflict with mothers/mothers-in-law
	-Perceived mismatching of social and emotional support
Adverse childhood experiences	-Neglect, obedience and strict discipline
	-Lack of positive parental role models during childhood
	-A positive and healthy intimate relationship

### Intensive mothering

The popular notion of ‘intensive mothering’ and childhood development theories that the participant women have learned through Chinese digital media have taken the dominant role in guiding their ideals of ‘being a good mother’. Most mothers in the study were determined to sacrifice their own comfort and needs to give the best to their children. During the early postpartum period, in particular, one of the biggest challenges for new mothers was to maintain exclusive breastfeeding. Almost every new mother was firmly convinced that exclusive breastfeeding for a longer period was crucial for developing the bond between the infant and the mother.

*You’ve heard a lot from people around you and social media about how attachment is important for children’s long-term development. I know I could have taken the ‘easy path’ of giving up breastfeeding because my nipples hurt, and I cannot get enough sleep, etc. But I believe that the best for my child is to bond with me through breastfeeding.*

--Qingying, has a 3-month-old son and a Dutch partner.

Qingying wanted not only to give the best to her child. Her way of speaking revealed the fear that if she could not reach the moral standards of modern mothering, that might ultimately translate into disadvantages for her child in future market-driven competition. When women experienced a discrepancy between the reality of mothering and the idealized version of intensive mothering, maternal guilt, stress, and anxiety all rose.

*I had doubts concerning my maternal abilities and had blamed myself for not trying hard enough to maintain exclusive breastfeeding for a longer time. After falling into the circle of being anxious, self-blame and guilt, I started to consult with my midwife. Even though her words comforted me, bottle-feeding is not good enough for my daughter.*

--Liangjing, has a 9-month-old daughter and a dutch partner

Despite the advice they received from Dutch midwives, many women like Liangjing, perceived the Dutch infant formula as second best. It indicated they failed to provide the best to their children according to the notion of ‘intensive mothering’ and therefore created maternal guilt. When a Chinese mother decided to take the ‘easy and comfortable’ path serving her own needs, she felt she was ‘incompetent’ (*bu chenzhi*) and falling short of ideal motherhood.

### Mothering and ambivalence: what parenting style is the best for my children?

*Based on my observation and talks with Dutch neighbors, I think most Dutch parents follow the principle of natural growth. Children will grow and thrive as long as we provide security and love.*

--Liangjing, has a 9-month-old daughter and a dutch partner

Liangjing described her first impression about Dutch parenting when she was asked about raising children in transnational context. Based on interactions and communications with Dutch parents, almost all participating Chinese mothers believed that Dutch parenting is a relaxed parenting style which is seen as ‘accomplishment of natural growth and development’. They reported experiencing an ongoing internal process of integrating mothering values from both Chinese and Dutch parenting ideals. Their narratives also showed mixed feelings concerning both the ‘intensive mothering’ as well as the Dutch ‘relaxing and happy’ parenting notion. Chinese migrant mothers feared that the Dutch parenting approach may lack goal orientation and a focus on childhood development for a promising future. On the other hand, they also noticed that the Dutch parenting approach offers an alternative path of nurturing a happy childhood, in contrast to the overscheduled style of childhood prevalent in urban China.

*How much does a child know? I make decisions for him because I am responsible for developing his talents for his better future. We [me and my husband] wish our son to have both the enjoyment and enough knowledge and skills through various activities such as playing an instrument, sporting, and off school classes.*

*--* Zhuyun, has a three-year-old son and a Chinese partner.

The term ‘opportunity’ (*Ji yu*) was embedded in almost every conversation with Chinese mothers. The middle-class Chinese mothers aimed at cultivating their children’s talents and preparing them to face life’s challenges in order to ensure better future opportunities, not only in the Netherlands, but also in a rapidly changing society in contemporary China. In this regard, the ‘good mothering’ ideal should result in flexibility and freedom for their children to pursuit their own interests as well as the necessary advantages to win in the global competition for talent. At the same time, Chinese mothers revealed their stress and anxiety concerning achieving two incompatible goals of parenting: to have a happy and natural childhood and to win in the international education and career competition. The distress they experienced ultimately reduced their abilities to cope with daily matters in early parenting.

### Perceived inadequate and mismatching social support from mothers and mothers-in-law

Most Chinese migrant women in our study received postpartum care ‘doing the month’ (*zuo yuezi*) from their own mothers or mothers-in-law, a hired Chinese maternity maid (*yuesao*) or, in some cases, they received mixed support. Despite the fact that social support is another key factor identified by many existing studies that may smooth the transition of motherhood, most women in our study experienced having their mothers or mothers-in-law as a burden.

*This is the battle you can never win. Grandparents represent the tradition that cannot be adjusted. Even though they do not know the reasons behind their certain beliefs, the tradition must be followed and carried out for the greater good of the extended family.*

-- Yao, 36 years old, has a 2-year-old daughter.

*I wanted some sympathy and emotional support from my mum about my pain, struggles and frustrations as a first-time mother. But she can barely understand my feelings. There was a huge mismatch between giving and demanding. A good rest is important for me, so I plan to give infant formula to my son during the night. But my mum thinks I am simply lazy and not trying hard enough for the best for my son.*

--Lele, has an 8-month-old son.

The intergenerational differences in values and beliefs were manifested explicitly in the early postpartum period. The tension between Chinese women and their own mothers and mothers-in-law originated from the fundamental rift between traditional Chinese mothering and modern practices of ‘intense mothering’ based on scientific knowledge. Doubts and concerns centered around the topic of traditional practices for infants’ health and new mothers, such as the following: ‘new mothers are not allowed to walk outdoors; ‘to have no coffee, tea, and spicy food during the breastfeeding period’; ‘to drink oily chicken soup every day’, etc. The term ‘forbearance’ (*yinren*) was often used by participating women to describe their early postpartum life during which they tried devoting everything to meet certain traditional mothering values of their mothers and mothers-in-law. The frustrations of constant arguments with their own mothers and mothers-in-law over the authority of childcare have exhausted new mothers. The unpleasant relationships with their own mothers and mothers-in-law, and inadequate practical and mental support were major contributors to maternal stress and anxiety that were, in turn, associated with the development of peripartum depression.

### Adverse childhood experiences & mental well-being

Exposure to adverse childhood experiences is known to increase the risk of psychological distress and poor maternal mental health that can result in less responsive maternal behaviors. In our study, there were 12 participating Chinese women who recalled adverse childhood memories and emphasized how much these experiences affected their development of becoming sensitive and responsive caregivers. The struggles in their childhood included neglect, obedience, and strict discipline, being constantly under pressure due to the comparison with peers and having little autonomy concerning personal interests and needs. Some of these participating women stated that they had received little warmth in their family of origin and felt flawed, unloved, and unwanted in childhood. These women had experienced feelings of frustration, guilt, sadness, depression and sometimes anger in the transition into motherhood, because they lacked positive parental role models in their own childhood. Their narratives indicated that growing up without a sensitive and emotionally warm parent could cause long-term disturbances in the attachment of mothers to their newborns.

*During pregnancy, old memories and images of my childhood flashed back. I felt that I was the unwanted and unloved one because I was not a boy who they wanted. I felt clearly that my daughter needed a safe and stable environment and that I was there for her to avoid repeating my own tough childhood. Many times, the frustration of failing to be a sensitive and caring mother made me feel hopeless and depressed.*

-- Xuanxuan, has a one-and-a-half-year-old daughter and a Dutch partner.

Furthermore, Chinese women’s perceptions of little emotional warmth in their own childhood could hinder the development of mother-infant bonding. These mothers were completely convinced that it was their obligations to break the cycle of poor parenting behaviors by their parents and to create a warm, empathetic, and responsive parenting environment for their own children. Nevertheless, many of them were struggling to transform unpleasant childhood memories into positive mothering values and practices. In this regard, these women perceived themselves as less competent mothers which, in turn, increased the risk of developing peripartum anxiety and depressive symptoms.

### Transforming the hardship from adverse childhood memories through positive intimate relationships

Despite the vulnerability and difficulty in motherhood due to adverse childhood experiences, we have also seen that a smooth and successful transition into motherhood can have a positive influence on a woman’s view of herself, her role in her marriage, and her maternal role. Some of the participating Chinese women have contemplated and attempted to revisit those difficult memories with great effort to make sense of them for the purpose of functioning as competent mothers.

*When my son was crying, I wanted to leave him alone in the room, locked the door and walked away. At such difficult moments, my man [her husband] would calm him down in his arms with soft voices and smiles. And he never blamed me. I cannot imagine how to go through the darkest time [struggling against postpartum depression] without my man.*

-- Lele, has an 8-month-old son and a Dutch partner.

Most Chinese women stated that they admired the relationships of their Dutch partners with their parents and considered these parents as ideal role models even before they decided to become mothers. Those women observed and perceived several key values in Dutch intimate relationships and families that they wished to have in their own families: love, care, respect, support, appreciation, and empathy. Looking up to those Dutch role models has helped Chinese mothers to realize that parenting and family life could also be characterized by warmth and empathy. Furthermore, a positive intimate relationship with one’s partner functioned as a protective factor by counteracting the uncertainty, stress, and anxiety in early motherhood. The support that mothers received from their partners improved maternal self-confidence and efficacy, increase feelings of happiness, and enhance maternal and mental well-being.

## Discussion

In our study, Chinese women have explored and developed the notion of modern mothering within the ‘public sphere’ on Chinese social media and the ‘private sphere’ within their local Chinese and Dutch social networks. These transnational sociocultural contexts have enabled Chinese women to reorient themselves in migrant motherhood, while two opposing parenting cultures have placed them in a dilemma of either being inadequate according to the modern Chinese ‘intensive mothering’ standard or being over-worried regarding the comparably relaxed and responsive Dutch norm. Despite the ambivalence towards different mothering values, these Chinese migrant women made best use of resources and information in transnational contexts to navigate what motherhood means to them as migrant mothers. In addition, Chinese mothers have experienced the fear for themselves of ‘failing parental responsibilities’ and for their children of ‘falling behind peers’ in the fierce competition for educational and career opportunities.

Through exploring the close relationships of Chinese migrant women, we further investigated the role of emotional support, various needs from social networks and their preferred ways of receiving help. Unwanted practical and emotional support from and interference by women’s mothers and mothers-in-law were viewed as burdens which often offset benefits of the assistance women received [27]. The tension and conflict when interacting with women’s mothers and mothers-in-law formed an accumulation of stress and anxiety. In the case of adopting the peripartum rituals of ‘doing the month,’ different childrearing values and interpersonal issues of autonomy triggered women’s perceptions of loss of control, increasing the susceptibility to develop symptoms of peripartum anxiety and depression which was in line with a previous study [13].

In contrast to previous studies showing that low economic status is a risk factor for developing peripartum depression, our participants were well-educated middle-class women who have high expectations of emotional and social support from their families and friends, but who were not in need for financial assistance. However, they became sensitive to changes in the transition into motherhood due to the unmet self-expectations regarding the pursuit of a good quality of life and a happy motherhood. Those well-educated middle-class migrant women were also overwhelmed by the increasing challenges and additional difficulties while acclimatizing to the host society. From this perspective, the advantages of their educational and economic background do not necessarily translate into a lower risk of developing peripartum depression.

In addition, the authors observed that some Chinese women with adverse childhood memories experienced additional difficulties during their transition to motherhood and stressed that they had to overcome considerable challenges in order to navigate the novel tasks of motherhood due to a lack of positive role models during their childhood. Confirming existing findings, we also found that supportive co-parents can help buffer against the potential negative impacts of adverse childhood memories [28]. The majority of Chinese mothers have asserted the positive changes in their maternal confidence and self-efficacy through warm, patient, and responsive support from their Dutch partners as well as observing their partner’s sensitive and responsive parenting styles. Furthermore, a healthy and nurturing intimate relationship with one’s partner can enhance maternal resilience and offer protective benefits against depressive symptoms.

### Limitations

All participants in our study were highly educated, middle-class, and married women. Therefore, our findings cannot be generalized to all Chinese migrant women in the Netherlands. Due to restrictions related to COVID-19, researchers lacked the observation of daily mothering practices. The conflicts with women’s own mothers and mothers-in-law and the support received were described by the women who were interviewed without field observations. Also, this study has not investigated the views of Chinese women’s partners regarding the topic of a healthy intimate relationship.

## Conclusion

In conclusion, the current study suggests that well-educated middle-class Chinese migrant women in the peripartum period are at risk of developing peripartum depression due to unmet self-expectations regarding the pursuit of a happy motherhood, the absence of desired social and emotional support, and adverse childhood experiences. Fostering nurturing intimate relationships and ensuring adequate social support in the host society have mitigated recollections of their adverse childhood experiences. Overall, our findings can provide valuable insights to interdisciplinary audiences, including general practitioners, midwives, and mental healthcare professionals, regarding the risk factors associated with peripartum depression among well-educated middle-class Chinese women in transnational contexts. The specific vulnerabilities of middle-class migrant women globally should be considered and distinguished from other subgroups. In addition, tailored intervention programs during the peripartum period for middle-class (Chinese) migrant women could support breaking the cycle of adverse childhood experiences and promote maternal bonding and resilience. To improve the mental healthcare seeking behavior of these women, healthcare professionals should receive training in culturally sensitive care skills, enabling them to address the specific needs of well-educated migrant women in mental healthcare services.

Future research on the experience of the transition to parenthood by both mothers and fathers is crucial to gain a comprehensive understanding of how changes in gender roles have shaped the roles of parents in transnational families. Urgent further research is also needed to determine whether targeted interventions for women who have both adverse childhood experiences and mental health issues can effectively prevent or reduce the impact of adverse childhood experiences on the development of peripartum depression.

### Electronic supplementary material

Below is the link to the electronic supplementary material.

Supplementary Material 1

## Data Availability

Quotes from the interview contents are published in this article. Full transcripts are not publicly available. Transcripts are available from the corresponding author on a reasonable request.
