# Supplementary material for: Experiences of peripartum depressive symptoms among Chinese middle-class migrant women in the Netherlands: a qualitative study of migrant motherhood
Source: BMC Pregnancy Childbirth. 2023 Sep 5;23:638. doi: 10.1186/s12884-023-05957-z (PMC10481590; doi:10.1186/s12884-023-05957-z)
Supplement: Supplementary file 1 — Supplementary Material 1 [file 12884_2023_5957_MOESM1_ESM.docx]

**Supplement file 1: The interview topic guide (English)**

**Semi-structure in-depth interview topic guide for Chinese migrant women**

**Brief introduction**

Becoming a mother is a big and happy life event in every woman’s life. Sometimes this journey can also be worrying, cause anxiety, make you upset and involve other emotional changes. As a migrant for a mother to be, adapting to a new environment could be difficult as well. In the interview, I would like to ask you questions regarding to your latest pregnancy and postpartum experiences on the following topics:

**Background questions**

Could you briefly describe your background, such as the place where you were born and grew up, at what age you moved to Netherlands, current family living conditions (whether also living together with own parents)

**Emotional feelings and changes**

1. Could you describe how your body and mind feel now in general? Are there any different feelings compared with the last month before giving birth? What are the differences? Could you try to remember whether there have been any moments of mood swing since the beginning of pregnancy until now? How did you feel during such mood swings? (anxious, nervous, upset, sad, disturbed, depressed, angry, panic etc.) What happened during those moments?
2. What are the big changes and challenges for you of becoming a mum? How do you feel about it? Do you feel you can handle it?
3. What do you think are the reasons for those feelings? Do you think those will go away after a while doing nothing about it?

**Emotional support and sources**

1. What did you do when you are/were experiencing those feelings? Did you try to talk to someone about it? Was there anyone close to you around? Who do you feel comfortable to talk to? Do you think it helps after? Have you ever felt you are far away from your family and old friends, which might make you feel lonely? Have you ever considered to talk to health professionals or to seek other ways of support?

**Social support in daily life and sources**

1. From the beginning of pregnancy till now, especially during “doing the month” period, who is providing you support in daily life? What kind of practical support do you need? (Did your parents or parents-in-law come from China to stay with you for a while?) What do they offer? How do you feel? about this support? Does it match your expectations? Is it perhaps in some sense too much or too little?
2. How’s the relationship between you and your partner or family members (your own parents and parents’ in-law)? Are there any marital or family conflicts, such as different views on ways of childrearing or ineffective communication? If so, could you elaborate more about it, such as in what condition/situation? How do you handle these issues and does this lead to positive outcomes after these situations?

**Knowledge, resources & trust**

1. During your pregnancy till now, what are your sources to learn knowledge and updated information about pregnancy, newborn and others? (such as friends and family, books, APPs, social media channels, peer groups in WeChat etc.) Which sources do you trust and listen to the most? Why? Are there any reasons why you didn’t join WeChat mum groups, if applicable?
2. Back in time, our parents were our main and most trusted knowledge sources about parenting. What do you think this is nowadays? How’s the situation in your family or your case? What are the reasons?

**Relations and experiences with Dutch maternal health care services**

1. Could you describe your experiences with the Dutch maternal health care system and providers in general? What kind of support did you receive from Dutch health care practitioners? What were your expectations at the beginning? How do/did you experience your relationship with your midwife? Which parts you feel more satisfied and not satisfied or even unhappy about? Have you tried to communicate with them if you feel unsatisfied? Any better and improvement?
2. Try to imagine, do you think your whole experience will be better if you were in China? Based on what you know or heard, what are the differences in services between China and NL? Which parts do you think are provided better by the Chinese system and what does the Dutch system provide better?

**Closing section**

This is the end of our interview. Thank you very much for your contribution. Is there anything you would like to add or ask? If you are interested, the interview transcripts can be shared with you and you are allowed to correct mistakes in it. Your help would be very much appreciated.

**The interview topic guide (simplified Chinese)**

**针对中国移民女性半结构深度访谈指南**

**简介**

成为母亲是每个女性一生中幸福的大事。有时，这一历程也会让人担忧、焦虑、不安，可能涉及其他情绪变化。作为一个移民的准妈妈或者新手妈妈，适应新环境也是一件困难的事情。在访谈中，我想就以下话题向您提问，了解您在怀孕期间和产后的经历：

**背景资料**

您能否简要描述一下自己的背景，例如出生和成长的地方、移民到荷兰的年龄、目前的家庭 生活状况（例如是否还与自己的父母住在一起）？

**关于情绪的变化和感受**

1. 您能描述一下您现在身心整体的感受吗？与分娩前的最后一个月相比，有什么不同的感受吗？在那些地方有哪些不同？您能试着回忆一下，从怀孕开始到现在，是否有过情绪波动的时候？在这种情绪波动时您有什么感觉(例如焦虑、紧张、烦躁、悲伤、不安、抑郁、愤怒、恐慌等）？在那些时刻具体发生了什么？
2. 成为母亲给您带来哪些巨大的变化和挑战？您对此有何感受？您觉得自己应对的如何？
3. 您认为产生这些感觉的原因是什么？如果不采取一些措施，您认为这些感觉会在一段时间后消失吗？

**关于情感支持和来源**

1. 当您有这些感觉时，您都做了什么？您有没有试着向别人倾诉？您身边有跟您很亲近的人吗？您觉得和谁倾诉更舒服更自在？您认为倾诉之后会有帮助吗？您是否曾觉得因为自己远离在中国的家人和故友感到孤独？您是否考虑过与一些专家交谈或寻求其他方式的支持？

**日常生活的社会支持和来源**

1. 从怀孕开始到现在，特别是在 "做月子 "期间，谁在日常生活中为您提供主要的支持？您需要哪方面的更实用的帮助和支持？(您的父母或岳父母是否从中国来荷兰陪伴您一段时间？） 他们提供了哪些帮助和支持？您对这些支持感觉如何？是否符合您的期望？从某种意义上说，您感觉获得的帮助和支持是太多还是太少？
2. 您与伴侣或家庭成员（您的父母和岳父母）之间的关系如何？是否存在婚姻或家庭矛盾，如对抚养子女的方式有不同看法或沟通不畅？如果有，请详细说明，例如在什么情况下？以及您是如何处理这些问题的？

**知识信息，来源以及信任**

1. 从怀孕到现在，您从哪些渠道了解到有关怀孕、新生儿及其他方面的知识和最新信息？(亲朋好友、书籍、手机APP、社交媒体渠道、微信群等）您最信任和最常听取哪些信息来源？为什么？如果您没有加入微信妈妈群，可以分享一下原因吗？
2. 在过去，父母是我们最主要、最值得信赖的育儿知识来源。您认为现在的情况如何？您的家庭或您的情况如何？原因是什么？

**与荷兰孕产妇医疗服务的关系和体验**

1. 您能否描述一下您在荷兰孕产妇医疗保健系统和医疗服务提供者那里的经历？荷兰医护人员为您提供了哪些支持？您最初的期望是什么？您与助产士之间的关系如何？您对哪些部分觉得比较满意和不满意甚至不高兴？如果您觉得不满意，您是否尝试过与她们进一步沟通？她们有没有更好地改进服务？
2. 您试着想象一下，如果您身在中国，您觉得您的整个孕期到生产的经历会更好吗？根据您了解或听说的情况，中国和荷兰的服务有哪些不同？您认为中国系统提供的哪些服务更好？荷兰系统提供的哪些服务更好？

**尾声**

我们的访谈到这里就结束了。非常感谢您的分享。您还有什么要补充或询问的吗？如果您有兴趣的话，我们可以与您分享访谈记录，您可以纠正其中的错误。非常感谢您的帮助。
